# Novel quantification of regional fossil fuel CO_2_ reductions during COVID-19 lockdowns using atmospheric oxygen measurements

**DOI:** 10.1126/sciadv.abl9250

**Published:** 2022-04-22

**Authors:** Penelope A. Pickers, Andrew C. Manning, Corinne Le Quéré, Grant L. Forster, Ingrid T. Luijkx, Christoph Gerbig, Leigh S. Fleming, William T. Sturges

**Affiliations:** 1 Centre for Ocean and Atmospheric Sciences, School of Environmental Sciences, University of East Anglia, Norwich NR4 7TJ, UK.; 2 National Centre for Atmospheric Science, University of East Anglia, Norwich NR4 7TJ, UK.; 3 Department of Meteorology and Air Quality, Wageningen University and Research, 6700AA Wageningen, the Netherlands.; 4 Department of Biogeochemical Systems, Max Planck Institute for Biogeochemistry, Jena, Germany.

## Abstract

It is not currently possible to quantify regional-scale fossil fuel carbon dioxide (ffCO_2_) emissions with high accuracy in near real time. Existing atmospheric methods for separating ffCO_2_ from large natural carbon dioxide variations are constrained by sampling limitations, so that estimates of regional changes in ffCO_2_ emissions, such as those occurring in response to coronavirus disease 2019 (COVID-19) lockdowns, rely on indirect activity data. We present a method for quantifying regional signals of ffCO_2_ based on continuous atmospheric measurements of oxygen and carbon dioxide combined into the tracer “atmospheric potential oxygen” (APO). We detect and quantify ffCO_2_ reductions during 2020–2021 caused by the two U.K. COVID-19 lockdowns individually using APO data from Weybourne Atmospheric Observatory in the United Kingdom and a machine learning algorithm. Our APO-based assessment has near–real-time potential and provides high-frequency information that is in good agreement with the spread of ffCO_2_ emissions reductions from three independent lower-frequency U.K. estimates.

## Teaser

“Atmospheric potential oxygen” is used to quantify fossil fuel CO_2_ reductions from COVID-19 U.K. lockdowns in 2020–2021.

## INTRODUCTION

Fossil fuel combustion and industrial processes are responsible for the majority of anthropogenic carbon dioxide (CO_2_) emissions, more than 70% of which are emitted from cities and urban areas (*1*). Despite their critical importance, our ability to evaluate reported emissions and to monitor and inform on the effectiveness of emissions reduction policies over the coming decades is currently limited (*2*, *3*). This limitation was recently highlighted by the 2020–2021 pandemic of the coronavirus disease 2019 (COVID-19). To mitigate the spread of the virus, many countries implemented social distancing measures at national or regional scales, resulting in sudden and severe temporary reductions in emissions of CO_2_ from fossil fuels (ffCO_2_) (*3*–*6*) and anthropogenic air pollutants (*6*–*8*). While numerous studies have successfully reported on air pollutant COVID-19 reductions as observed from atmospheric measurements (*6*–*8*), determining ffCO_2_ COVID-19 reductions in the atmosphere has been substantially more challenging, owing to the large variations in atmospheric CO_2_ caused by terrestrial biosphere fluxes (*9*).

The Paris Agreement invokes an increased imperative to report anthropogenic CO_2_ emissions accurately at the country and subcountry scale with transparency and consistency (*2*) and to develop methods for independent evaluation (*10*, *11*). Currently, anthropogenic CO_2_ emissions are self-reported to the United Nations Framework Convention on Climate Change using an indirect “bottom-up” approach, based primarily on energy statistics and emission factors, and an agreed methodology (*12*); however, large inconsistencies in bottom-up approaches have been reported, arising from inaccuracies in energy statistics and/or emission factors (*13*–*15*).

The Global Carbon Budget 2020 (*5*) provided a detailed assessment of the impact of COVID-19 on ffCO_2_ emissions during 2020 at global and regional scales, based on a range of bottom-up assessments, including those that also incorporate recently available activity and mobility tracking data, such as the Carbon Monitor product (*4*) and the Priestley Centre estimate (*6*). Relative changes in emissions during 2020 from this suite of bottom-up estimates reveal large inconsistencies in many regions, such as the EU27 (the 27 member states of the European Union), for which the reductions in ffCO_2_ are 9.6% [University of East Anglia estimate, hereafter “UEA” (*3*)], 12.9% (Priestly), 7.1% (Carbon Monitor), and 17% (Global Carbon Budget) (*5*). Furthermore, since the suite of Global Carbon Budget bottom-up estimates is not available in real time, year-to-date projections were included, on the basis of forward extrapolation of emissions reductions to the end of the year 2020, instead of using emissions estimates based on actual lockdown measures (*5*). Nevertheless, this comparison of methods gives an indication of the uncertainty in regional estimates based on indirect proxies.

Attempts to detect and quantify COVID-19–associated ffCO_2_ emissions reductions using more direct “top-down” methods, based on atmospheric measurements and modeling, have largely been unsuccessful so far, particularly at regional and country scales. In the United Kingdom, a study based on atmospheric CO_2_ data from the Deriving Emissions linked to Climate Change network (*16*) found that COVID-19 signals will only be detectable in daily CO_2_ mole fractions after at least 33 months of sustained emissions reductions (*9*). Another study, using atmospheric CO_2_ data from the European Integrated Carbon Observing System network (https://icos-cp.eu/observations/atmosphere/stations) and the Stochastic Time-Inverted Lagrangian Transport model (STILT) (*17*), was unable to detect COVID-19–related reductions in ffCO_2_ associated with the first wave of lockdown measures in Europe (https://icos-cp.eu/sc2020/abstracts#152). In East China, a study using satellite CO_2_ retrievals was also unsuccessful (*18*). In all of these studies, COVID-19 ffCO_2_ reductions were obscured by fluxes of CO_2_ between the atmosphere and the terrestrial biosphere, which are typically much larger than ffCO_2_ emissions. At Hateruma Island, Japan, atmospheric measurements of CO_2_ and methane (CH_4_) were used to infer COVID-19–related ffCO_2_ reductions, mostly from wintertime data (when biospheric activity is suppressed), and by assuming that biospheric-related variability in CO_2_:CH_4_ ratios was not different in 2020 compared to previous years (*19*).

At the urban scale, where the contribution of ffCO_2_ emissions relative to biospheric CO_2_ emissions is usually larger, detection of COVID-19 signals has been possible in some locations using observations from satellites. A reduction in ffCO_2_ emissions of 11.5% was detected in China during January to April 2020 (compared to the same months in 2019) using satellite-based nitrogen dioxide (NO_2_) observations and CO_2_-to-NO*_x_* emission ratios from a bottom-up estimate to formulate proxy ffCO_2_ observations (*20*); however, about half of the satellite grids were excluded from this analysis owing to the prevalence of natural emissions in the observations, which rendered the data unusable for ffCO_2_ quantification. In another study, CO_2_ measurements from a high-density low-cost sensor network in the San Francisco Bay Area were combined with satellite measurements of solar-induced fluorescence (a proxy for biospheric CO_2_ emissions) and a high-resolution bottom-up ffCO_2_ emissions prior within an atmospheric inversion framework. The authors found a 30% reduction in ffCO_2_ emissions during a 6-week period of the city’s “shelter-in-place” order, compared to the previous 6-week period (*21*). In both of these studies, the detection of a distinct COVID-19 signal was only made possible owing to (i) the use of proxy/“tracer” observations to separate the anthropogenic and natural contributions to the total atmospheric CO_2_ signal, (ii) the availability of high-resolution emissions or emission ratio information from bottom-up inventories, and (iii) the selection of urban-based measurement locations, where ffCO_2_ signals are comparatively larger [typically from 0 to 30 parts per million (ppm)] than signals at non-urban sites (usually less than 10 ppm).

In addition to satellite-based proxies/tracers, natural and anthropogenic signals in CO_2_ can also be separated with ground-based atmospheric measurements, using radiocarbon data (^14^CO_2_), carbon monoxide data (CO), or a combination of both (*22*–*29*). ^14^CO_2_, the current “gold-standard” ground-based ffCO_2_ tracer method, is a high-precision measurement that has been recently used to successfully provide a top-down assessment of ffCO_2_ emissions in the United States (*11*). The main limitations of using ^14^CO_2_ are twofold: First, it is currently only possible to measure atmospheric ^14^CO_2_ with high accuracy from discrete samples (i.e., noncontinuously, with relatively low temporal resolution), which are moreover expensive and laborious to analyze (*30*); second, in some regions, such as the United Kingdom, ^14^CO_2_ measurements can be severely influenced by CO_2_ emissions from gas-cooled nuclear power plants, which obscure ffCO_2_ signals in ^14^CO_2_ data (*31*, *32*). CO, a continuous high-frequency ffCO_2_ tracer that is easier to measure and is unaffected by nuclear power plant emissions, can also be used, either as an alternative to ^14^CO_2_ sampling or in conjunction with ^14^CO_2_; however, CO-based ffCO_2_ is limited by poor precision and accuracy, mostly arising from highly variable and inaccurate CO:ffCO_2_ emission ratio information, which is required for CO-based ffCO_2_ quantification (*24*, *25*). Despite these limitations, ground-based measurements are more precise and accurate than satellite-based measurements; conversely, satellite-based measurements provide higher spatial coverage than ground-based measurements. To date, very few studies have been able to use ground-based atmospheric measurements to provide a top-down assessment of COVID-19–related ffCO_2_ emissions reductions.

The rate of COVID-19–related emissions reductions during 2020–2021 was similar to the rate of long-term emissions reductions required by the Paris Agreement to reach net zero emissions and limit global temperature rise in the range of 1.5° to 2°C. COVID-19 has demonstrated that, despite the critical importance of ffCO_2_ emissions reductions for climate change policy, we do not currently have systems in place—either bottom-up, top-down, or a combined approach—to monitor and report ffCO_2_ emissions at global, regional, or country scales in near real time (*3*).

Here, we present a new ground-based measurement approach for quantifying the regional ffCO_2_ component of the atmospheric CO_2_ mole fraction (in parts per million) using atmospheric potential oxygen (APO) data. We demonstrate the potential of APO as a ffCO_2_ tracer by detecting and quantifying COVID-19 ffCO_2_ reductions in the atmosphere associated with the first two waves of the pandemic in the United Kingdom, using continuous data from the Weybourne Atmospheric Observatory (WAO) in the United Kingdom and a machine learning algorithm. The APO-based assessment we present here separates biospheric and anthropogenic signals in atmospheric CO_2_ with high frequency (e.g., daily or subdaily scales) and in near real time, which is an important first step toward robust quantification of absolute ffCO_2_ emissions using atmospheric data. Our approach does not quantify absolute emissions, but, with the use of machine learning, we are able to quantify relative changes in emissions using APO data, which represents a major achievement in top-down observation-based ffCO_2_ emissions quantification efforts. Using a combined APO and machine learning approach, we have detected a local 1.6-ppm reduction in daily-mean ffCO_2_ observed at WAO during March to July 2020 compared to the nonpandemic “counterfactual scenario” (i.e., compared to the expected ffCO_2_ during 2020 if the COVID-19 pandemic had not occurred), and a 1.3-ppm daily-mean reduction during November 2020 to January 2021. These two U.K. lockdown periods are separated by a period of recovery, from August to October 2020, characterized by little reduction in ffCO_2_. Our APO-based estimate is in good agreement with the spread of ffCO_2_ reductions determined from three independent bottom-up emissions estimates for the United Kingdom.

## RESULTS

### Calculation of ffCO_2_ from APO

**Fig. 1.**
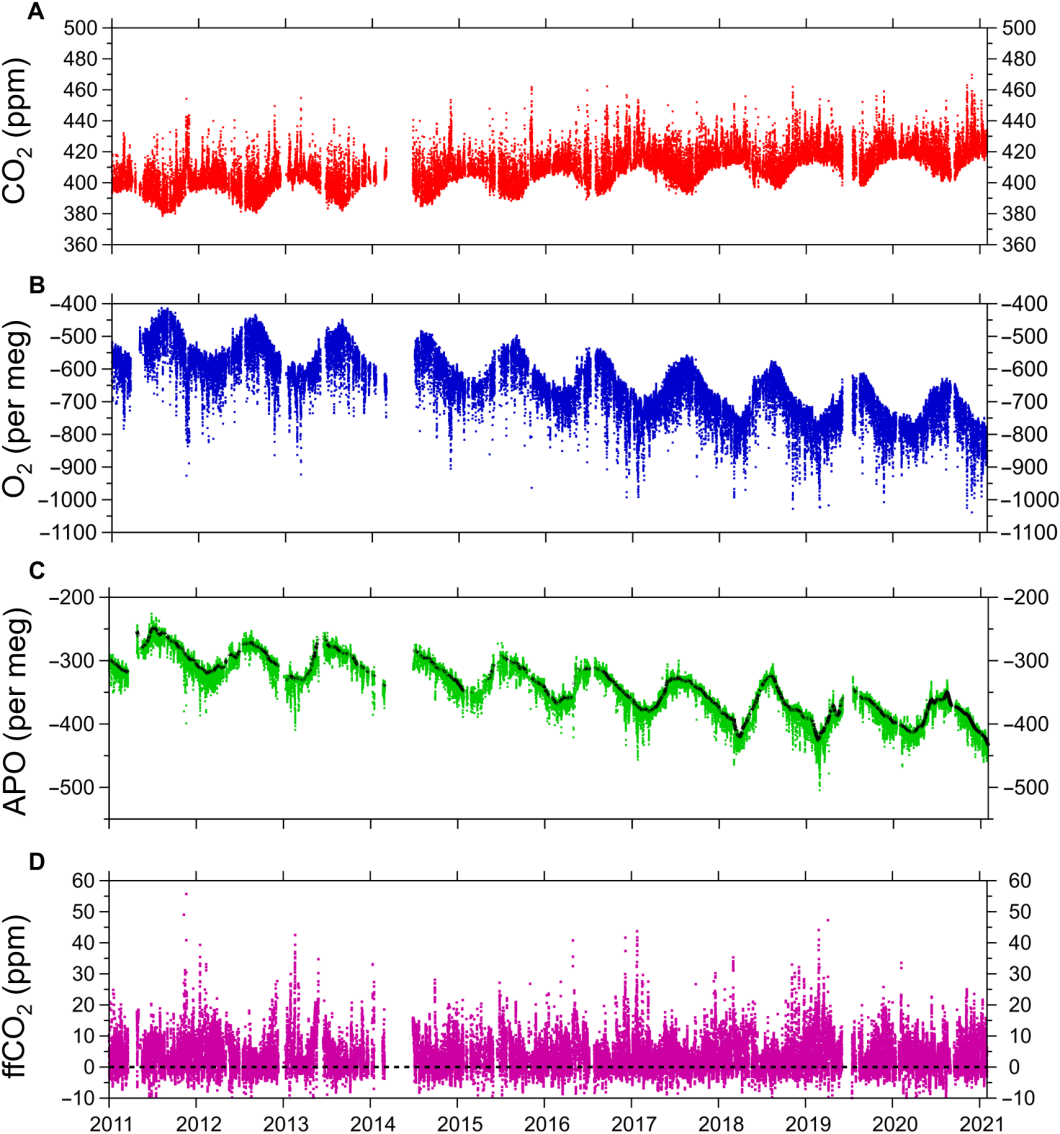
Hourly atmospheric CO_2_, O_2_, and APO observations and calculated ffCO_2_ from the WAO, 2011–2021. (**A**) Atmospheric CO_2_ in parts per million. (**B**) Atmospheric O_2_ in per meg units. A 1-ppm change in CO_2_ is equivalent to a 4.77–per meg change in O_2_ (*38*). (**C**) APO, also in per meg units. The black points in (C) are the statistically determined “baseline,” i.e., the APO_BL_ term in Eq. 1. (**D**) ffCO_2_, calculated from APO by removing the baseline signal in (C) from the APO observations and dividing by *R*_APO_ as in Eq. 1. The black dashed line denotes “zero” ffCO_2_, which is defined as the statistically determined baseline APO concentration. (A) to (C) show seasonality that is driven mostly by terrestrial biospheric processes (CO_2_ and O_2_) and oceanic processes (O_2_ and APO). Shorter-term variability in all panels is driven by diurnal processes, changes in meteorological conditions, synoptic-scale variability, and ffCO_2_ emissions. Gaps in the data are caused by instrument downtimes. *x* axis tick labels denote the beginning of the year shown.

APO is a tracer that combines oxygen (O_2_) and CO_2_ observations (APO = O_2_ + 1.1 × CO_2_) (*33*), where the value of 1.1 denotes the mean −O_2_:CO_2_ molar ratio of terrestrial biosphere-atmosphere exchange (*34*). APO is therefore, by design, invariant to terrestrial biosphere exchange processes. We calculate ffCO_2_ from APO, which we refer to as ffCO_2_[APO], according to

$${\text{ffCO}}_{2}[\text{APO}]=\frac{\text{APO}-{\text{APO}}_{\text{BL}}}{R_{\text{APO}}}$$ (1)

where APO_BL_ is the “baseline” APO value, which is determined statistically (see Fig. 1C and Materials and Methods), and *R*_APO_ is the molar ratio (*R*) of APO:CO_2_ for fossil fuel emissions, derived from an emissions database product (*35*) (see Materials and Methods for details). Both APO and APO_BL_ have units of “per meg,” rather than mole fraction units (such as parts per million) because O_2_ is not a trace gas and its mole fraction is therefore affected by small changes in other gases, such as CO_2_ (*36*). While APO has typically been used in the past to remove the influence of land biospheric exchange to isolate air-sea O_2_ and CO_2_ fluxes (*33*, *37*), here, we use APO to isolate fossil fuel emissions by subtracting a baseline that includes air-sea influences on APO (which mostly operate on long-term and seasonal time frames; see Materials and Methods). The numerator (APO-APO_BL_) thus isolates short-term anomalies in APO, from which ffCO_2_[APO] is determined. We convert the units of APO-APO_BL_ from per meg to parts per million equivalent by dividing by 4.77 (*38*). A full derivation of Eq. 1 is provided in appendix SA of the Supplementary Materials.

**Fig. 2.**
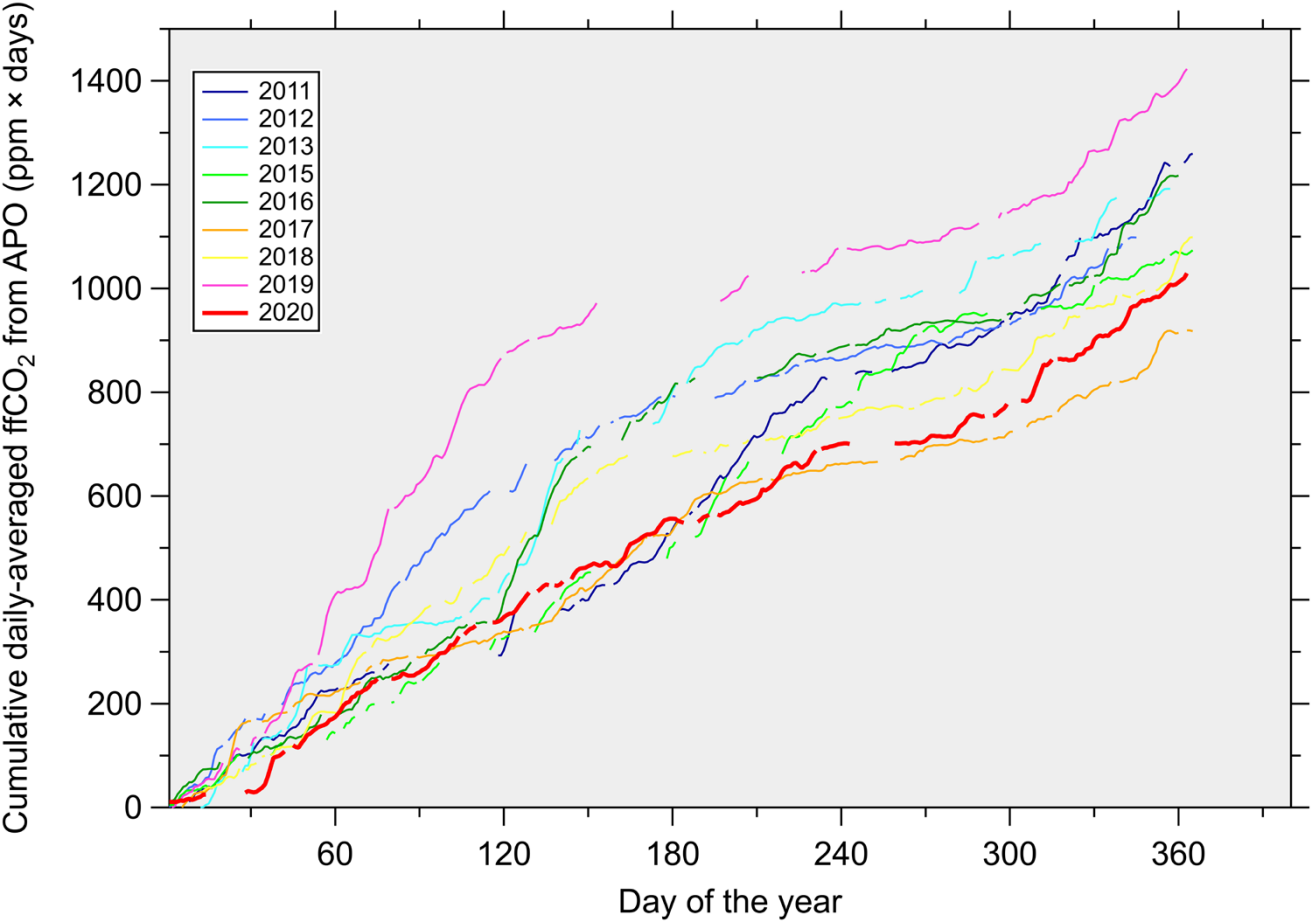
Cumulative daily ffCO_2_ from APO in parts per million × days observed at WAO. Nonpandemic years (2011 to 2019) are shown by the thinner colored lines, except for the year 2014, which is omitted because of large gaps in the data. The year 2020, during which the COVID-19 pandemic started, is shown by the thicker red line. The influence of gaps on the cumulative signals have been accounted for by adjusting the ffCO_2_ by the proportion of days that are missing data in each year. The 29 February has similarly been excluded where relevant, to allow a fair comparison between leap and nonleap years.

Figure 1 shows the atmospheric CO_2_, O_2_ and APO record from WAO in the United Kingdom, and the resulting ffCO_2_ calculated from APO. Because of WAO’s rural location on the north Norfolk coast, atmospheric transport variability exerts a substantial influence on the observed ffCO_2_ signal (figs. S1 and S2). Air masses arriving at the site from the North Sea and the Arctic Ocean are generally associated with lower ffCO_2_ compared to air masses arriving from the direction of southern England, the Midlands, or from the European continent. A cumulative plot of ffCO_2_ for each year during 2011–2020, shown in Fig. 2, reveals that there is no apparent difference in the total ffCO_2_ observed during 2020 compared to previous years because the dominating influences of wind direction and atmospheric transport on the ffCO_2_ signals at WAO have not been accounted for.

### COVID-19 ffCO_2_ detection

**Fig. 3.**
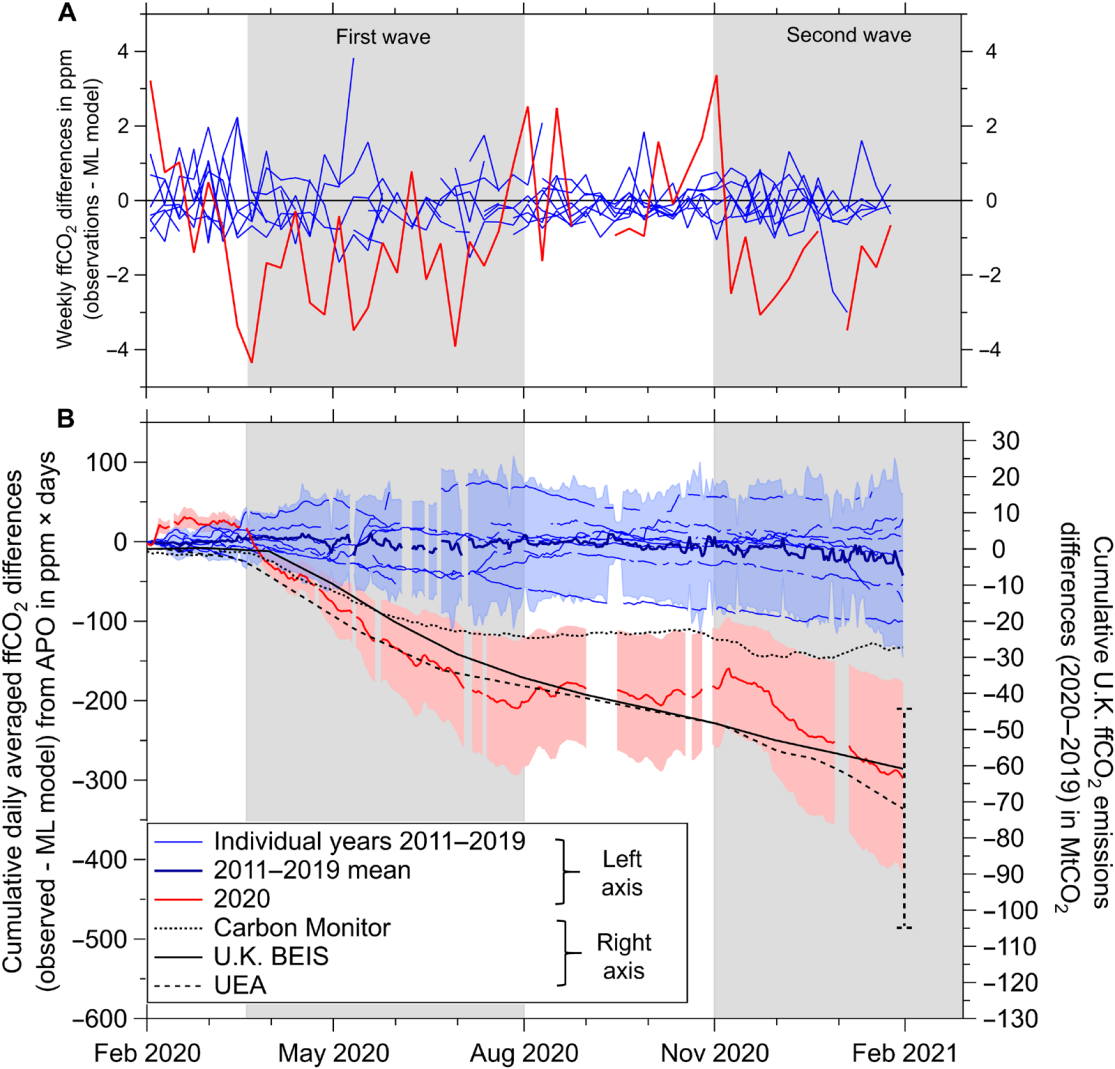
Reduction in WAO ffCO_2_ associated with COVID-19 lockdowns. (**A**) Differences in ffCO_2_ [as ffCO_2_ determined from APO minus modeled ffCO_2_ determined from a random forest machine learning (ML) algorithm], shown as weekly differences. The first and second U.K. COVID-19 waves are indicated by the gray background shading. Differences for the individual years 2011–2019 are show in blue. The period February 2020 to January 2021 is shown in red. All units are parts per million; *x*-axis major tick marks denote the first day of the month. Uncertainties are omitted from this panel for clarity. (**B**) Same as (A), but shown as cumulative daily-averaged ffCO_2_ in units of parts per million × days. The thick blue line indicates the 2011–2019 mean. Uncertainties are as follows: The blue shading is the ±2σ (95%) SD of the 2011–2019 mean, shown by the thick blue line, and represents the uncertainty of the training model (i.e., if the model performance was perfect, then the blue lines would all be zero), which, in part, arises from the long-term decreasing trend in U.K. emissions over the period 2011–2019; ffCO_2_ uncertainty for February 2020 onward is shown by the pale red shading and arises from the poorer performance of the predictive model relative to the training model (see the “Analysis of uncertainties” section for details). For comparison with our ffCO_2_[APO] detected COVID-19 signal, we also show 2020–2019 differences from three bottom-up U.K. emissions estimates (black lines) on the right-hand axis in units of MtCO_2_ (see Materials and Methods). Only the UEA value (black dashed line) includes an estimate of uncertainty, shown by the vertical error bar.

We have applied the APO method to detect and quantify the reduction in ffCO_2_[APO] associated with the U.K. COVID-19 lockdown restrictions, using an 11-year continuous APO dataset from WAO and a random forest machine learning algorithm (*39*) to account for the effects of weather and atmospheric transport processes on APO (Fig. 3). A suite of 10 independent variables with hourly time resolution (see Materials and Methods) was used to train the machine learning algorithm to model ffCO_2_ for the period 2010–2019. The algorithm was then used to predict the counterfactual case for February 2020 to January 2021, that is, the expected ffCO_2_ that would have been observed at WAO during this period had there been no pandemic. Weekly differences are shown in Fig. 3A, which indicate reductions in ffCO_2_ relative to the counterfactual (nonpandemic) prediction during periods when COVID-19 restrictions were in place.

The COVID-19 influence on ffCO_2_ detected at WAO is highlighted using the cumulative signal, shown in Fig. 3B, which accumulates differences in the short-term variability of the daily values. We find a sustained decrease in daily-mean ffCO_2_[APO] relative to the counterfactual (nonpandemic) prediction of 1.6 ppm from 20 March to 31 July 2020, coinciding with the first period of U.K. lockdown, a recovery period during August to October 2020 during which U.K. lockdown restrictions were eased and ffCO_2_[APO] increased slightly by 0.2 ppm, and a second sustained decrease in daily-mean ffCO_2_[APO] of 1.3 ppm from early November 2020 until the end of January 2021, during which a national lockdown was reintroduced. We deem these sustained signals to be caused by reductions in ffCO_2_ emissions within the footprint of WAO, where the term “footprint” denotes the sensitivity of measurements at WAO to emissions located upwind of the site location (*24*). WAO is influenced most by southwesterly winds, so the site predominantly captures ffCO_2_ signals from London and southern England over emissions from other wind sectors. WAO is therefore not representative of the United Kingdom as a whole (signals at WAO can also include emissions from continental Europe in addition to U.K. emissions under certain atmospheric conditions).

For the full year 2020, we find an overall mean reduction of 0.7 ppm. This estimate is higher than the expected global CO_2_ change associated with COVID-19 emissions reductions [which is ~0.3 ppm for an 8% reduction in 2020 annual emissions according to another study (*40*)], most likely because the U.K. drop in COVID-19 emissions was substantially larger than the global average (*41*).

### Comparison with independent estimates

Both the timing of the onset and the shape of the cumulative ffCO_2_ signal from APO (Fig. 3B) agree with three bottom-up estimates of the emissions decrease from COVID-19 lockdown measures for the United Kingdom, based on indirect activity data (black lines in Fig. 3B). The magnitudes of these estimates are not directly comparable because the ffCO_2_[APO] top-down signal is in units of parts per million × days and is not representative of the United Kingdom as a whole, while the bottom-up COVID-19 signals are in megatons of CO_2_ and are U.K. totals. In addition, our ffCO_2_[APO] reduction is relative to the counterfactual prediction for 2020, whereas the bottom-up estimates are relative to emissions for 2019. Nevertheless, for the period 1 February 2020 to 31 January 2021, we find a 23% reduction (range of 14 to 32%) from ffCO_2_[APO], compared to a 17% reduction from the U.K. BEIS (Department for Business, Energy, and Industrial Strategy) national inventory. Estimates based on proxy data give an 8% reduction from Carbon Monitor (*4*) and a 21% reduction (range of 13 to 30%) from the updated UEA estimate (*3*). Uncertainty ranges are not available for Carbon Monitor and U.K. BEIS estimates.

The spread in bottom-up estimates shown in Fig. 3B can in part be accounted for by differences in international aviation and shipping (IAS) emissions, which are included in the UEA estimate but are not included in the U.K. BEIS estimate. The Carbon Monitor estimate includes international aviation but not shipping. U.K. IAS emissions reductions resulting from the COVID-19 pandemic are estimated to be 17.3 MtCO_2_ in 2020 (*41*), which would bring the Carbon Monitor estimate closer to the UEA estimate but does not account for all of the offset. A similar adjustment to the U.K. BEIS estimate would shift it lower than the UEA value, so that even if IAS is included in all three bottom-up estimates, a range of ~30 MtCO_2_ would still persist. In addition, since only the UEA estimate includes an estimate of uncertainty, it is likely that the spread between bottom-up estimates would be substantially larger than this if uncertainties were available for the U.K. BEIS and Carbon Monitor estimates.

### Analysis of uncertainties

We will show in this section that it is not required to quantify uncertainty in ffCO_2_[APO] to calculate the uncertainty in relative ffCO_2_ emissions reductions from APO, but we nevertheless include detailed information in Materials and Methods on ffCO_2_[APO] uncertainties for reasons of transparency and completeness. In summary, ffCO_2_[APO] uncertainties are calculated for each of the terms in Eq. 1: the *R*_APO_ value for WAO, the statistically derived baseline uncertainty APO_BL_, and the uncertainty of the APO data themselves. Total hourly ffCO_2_[APO] uncertainty can thus be calculated by subtracting and dividing the absolute and relative errors according to the rules of error propagation.

For our COVID-19 analysis, while the ffCO_2_[APO] values could contain bias, either from inaccuracies in APO_BL_ or from an inaccurate *R*_APO_ value, we do not expect such bias, if it exists, to translate into error in our COVID-19–related relative ffCO_2_ emissions reduction estimate because the random forest algorithm is trained on similarly biased ffCO_2_[APO] data (and since we calculate observation-model differences, most bias should cancel). This assumption relies on our ffCO_2_[APO] error remaining the same during both the training and predictive steps. For the APO measurement data and APO_BL_, there is no indication in the diagnostic data that this is not the case: Measurement performance during 2020–2021 was similar to previous years, and there is no reason why the statistical baseline fitting routine should be less accurate during this time period than previously nor any evidence in the data to suggest that this is the case.

For *R*_APO_, a shift in this value associated with COVID-19 lockdowns would potentially bias our results because we used a mean *R*_APO_ value of 0.37 throughout the whole 2010–2021 period; however, it is unlikely that any COVID-19–related *R*_APO_ shift occurred, since bottom-up estimates have shown that the reduction in ffCO_2_ emissions is mostly in aviation and surface transport (*3*, *4*). The former should not have a large impact on our APO-based analysis because WAO is not situated near any airports. We do not expect the latter to substantially bias *R*_APO_ because surface transport emissions are predominantly from liquid-based fuels, with *R*_APO_ values of ~0.34, which sit approximately in the middle of the *R*_APO_ range between solid- and gas-based fuel types (*35*). A 36% reduction in surface transport, as found by Le Quéré *et al.* (*3*), would correspond to a bias in the *R*_APO_ value of +0.02, which would have a very small impact on ffCO_2_[APO]. In addition, we have calculated *R*_APO_ using the U.K. BEIS inventory (fig. S3), which shows a change in *R*_APO_ during the COVID-19 lockdown periods of only +0.01. During 2011–2019, *R*_APO_ does not change substantially except during 2012–2015, when the value changes by −0.05 because of reduced coal emissions. *R*_APO_ returns to its original 2011 value in 2016 due to increases in gas usage, which counteracts the previous influence of coal on *R*_APO_. Thus, there is no requirement to account for ffCO_2_[APO] uncertainties in our COVID-19 analysis, although these do exist (see Materials and Methods) and may need to be considered carefully in potential follow-up studies.

**Fig. 4.**
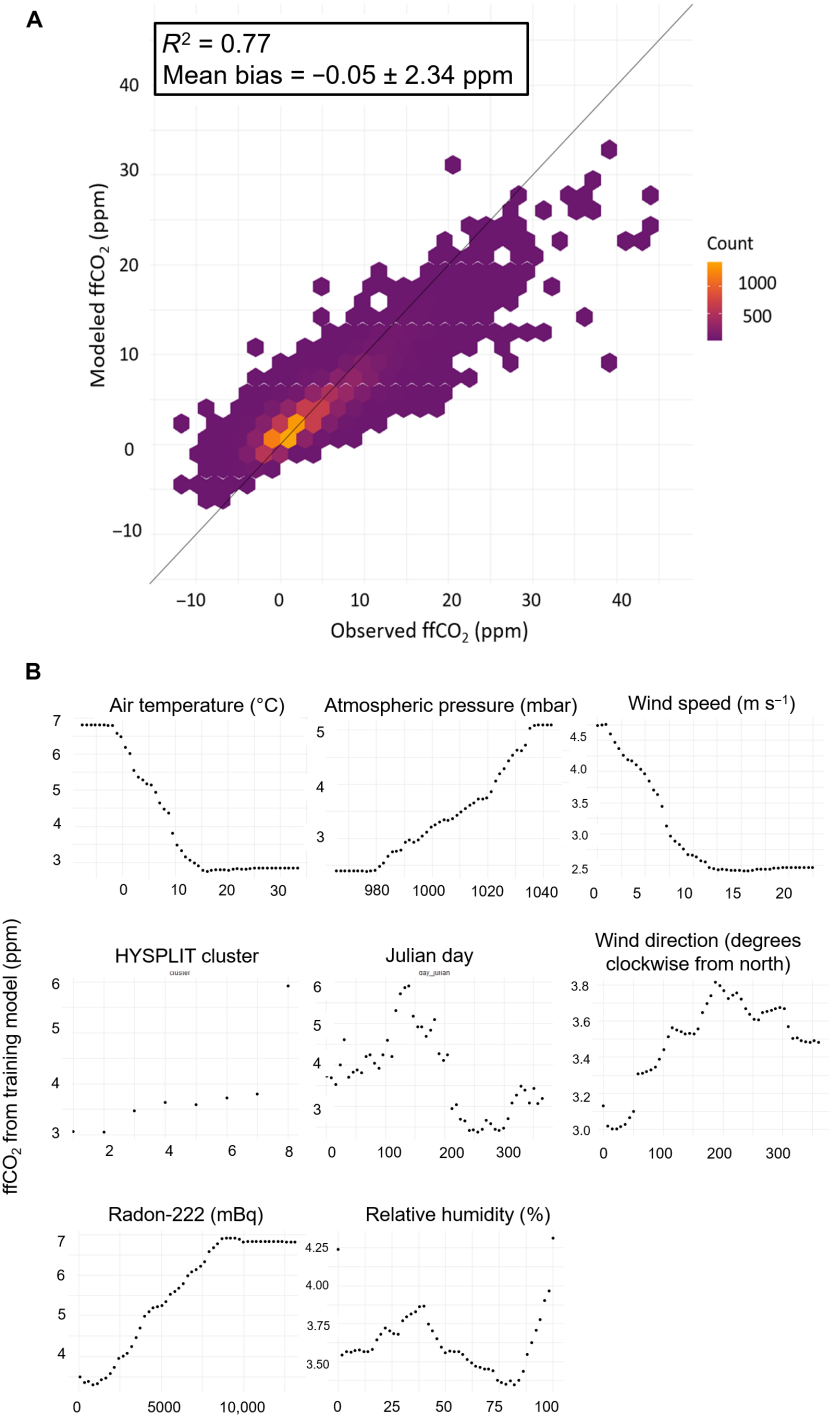
Evaluation of random forest machine learning model. (**A**) Scatter plot of hourly observed versus modeled ffCO_2_ from the random forest model (2010–2019 only), showing the mean of the differences ±1σ SD. The plot is created using data from the model test set only, which are withheld from model training. The black line represents a 1:1 relationship. Observed ffCO_2_ is calculated using the APO approach (see Materials and Methods). The model underestimates the true range of variability of the APO-based ffCO_2_ but generally performs well. A histogram of the differences is shown in fig. S4. (**B**) Partial dependence plot of the key independent variables of the trained random forest model. The plots show the relationship between each independent variable and modeled ffCO_2_ (from the trained model) and therefore provide insight into how variables are being used in the predictive model (*39*). See fig. S5 for the HYSPLIT cluster key.

It is, however, necessary to consider the performance of the machine learning algorithm and its associated uncertainty, which we assess as follows. First, we evaluate the performance of the model, as shown in Fig. 4. The model underestimates the true range of variability of the APO-based ffCO_2_ but generally performs well with a relatively small bias (−0.05 ± 2.34 ppm; see Fig. 4A and fig. S4). The impact of the imperfectly trained model performance on our ability to robustly detect COVID-19 reductions in ffCO_2_ is shown by the blue lines in Fig. 3B, which would all be zero if the trained model was perfect. The spread in these lines also includes the decreasing trend in U.K. emissions (−22% during 2011–2019, from BEIS data), which is not captured by the machine learning algorithm because bottom-up emissions are deliberately not used in the model training as a variable. By examining the partial dependencies of the independent variables (Fig. 4B), which shows the relationship between each variable and ffCO_2_ for the training model, we can also see whether the model performs as we would expect. We find that high ffCO_2_ at WAO is associated with lower air temperatures (during winter), higher atmospheric pressure and lower wind speeds (during more stable conditions), higher radon-222 activity (more influence from ground-based sources and/or lower planetary boundary layer height), and when clustered trajectories from the Hybrid Single Particle Lagrangian Integrated Trajectory (HYSPLIT) model (fig. S5) originate from the south, especially the southeast (i.e., from the European continent). Low ffCO_2_ at WAO is observed during opposite conditions, such as during the summer months and when the wind direction is northerly (from the ocean). A ranking of the importance of each independent variable for the model training is shown in fig. S6. These results collectively indicate that the trained machine learning model provides a physically realistic representation of ffCO_2_ at WAO.

Second, we recognize that the model prediction is less robust than the trained model, and an additional uncertainty (±40%; pale red shading in Fig. 3B) is assigned to the 2020–2021 counterfactual case to account for this. This ±40% model prediction uncertainty was estimated by quantifying the difference between the predicted counterfactual from a separate model that was trained on 2010–2018 data and used to predict the period 1 January to 31 December 2019 ffCO_2_, to the trained data from the original model for the period 1 January to 31 December 2019. Using the model to predict ffCO_2_ during previous nonpandemic years does not result in erroneous COVID-19–type signals, which should occur if the model prediction is consistently prone to overestimation.

Third, to ensure that the random forest prediction is not overly sensitive to the data at the beginning of the period (i.e., to so-called “end effects”), we examined ffCO_2_ reductions from a variety of predictions run with differing start dates (fig. S7). Although the choice of start date does have a small impact on the magnitude of the pandemic ffCO_2_ signals, all predictions still show similar patterns associated with the two COVID-19 lockdown periods with a period of lockdown easing during summer 2020. None of the differences are outside of the uncertainties, except for during the first few months, when the uncertainties are small. The sensitivity of the random forest prediction to other parameters, such as the *R*_APO_ value used to calculate ffCO_2_ from APO, and the stiffness of the APO baseline fit were also tested, but no notable differences were found.

### COVID assessment from WAO atmospheric CO_2_ data

**Fig. 5.**
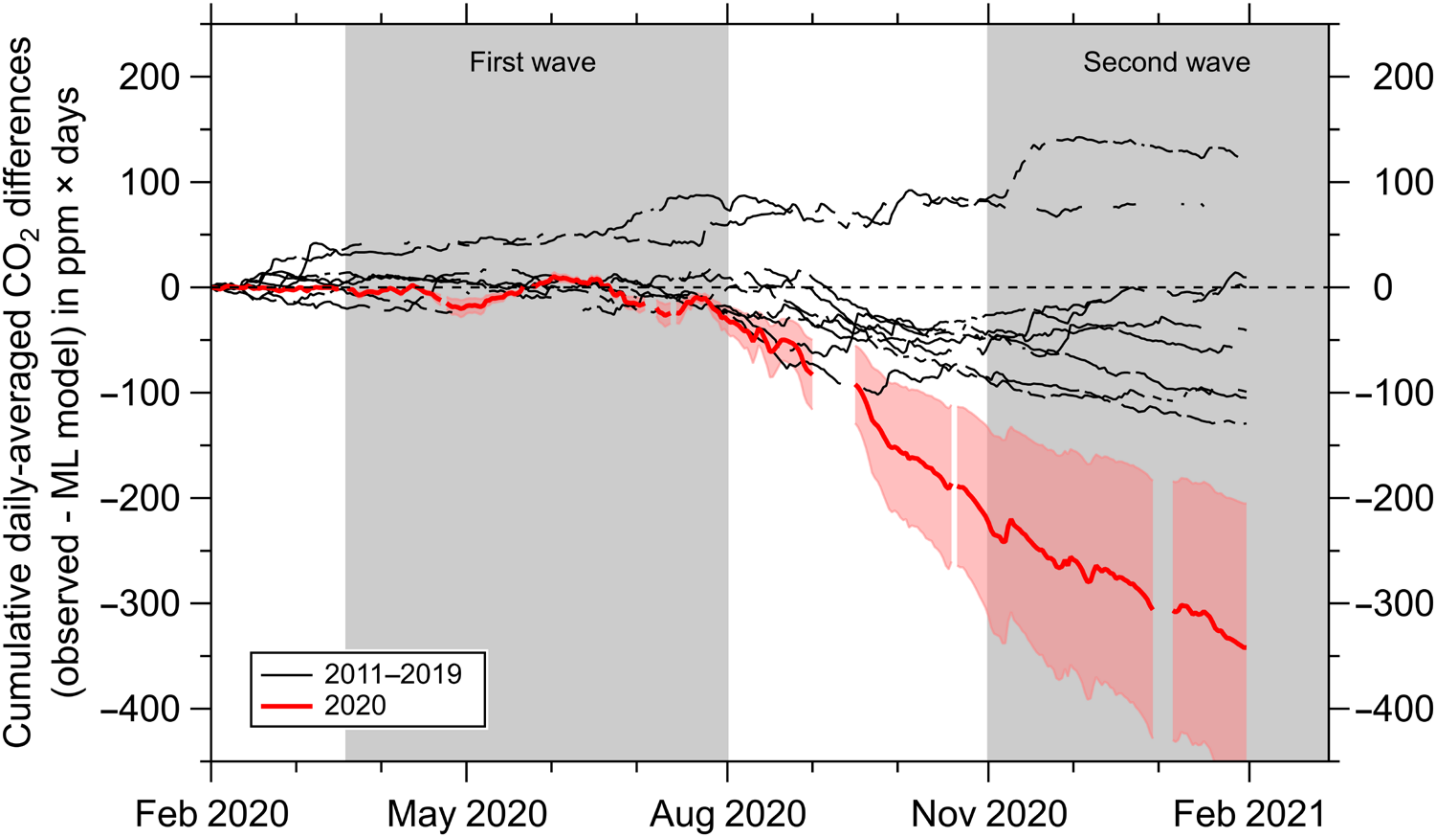
Results of the random forest prediction using atmospheric CO_2_ data. The years 2011–2019 are shown by the black lines. The year 2020 is shown by the red line, with ±40% uncertainty of the machine learning prediction indicated by the red shading. The uncertainty of the daily CO_2_ observations themselves is not shown, since this is extremely small (typical hourly uncertainties are less than ±0.1 ppm).

We also applied the random forest machine learning algorithm to WAO atmospheric CO_2_ data instead of APO data (Fig. 5) but found that a signal that could potentially be ascribed to ffCO_2_ reductions only emerges from about mid-September 2020 onward, 6 months after lockdown restrictions were first introduced. The timing of this potential signal is not consistent with the timing of the United Kingdom’s second lockdown, and it is not possible to separate the observed CO_2_ reduction into ffCO_2_ emissions reductions versus changes (either reduction or enhancement) in biospheric CO_2_ emissions. It is more likely that the initial reduction in CO_2_ during the late summer of 2020 is caused by an anomaly in biospheric CO_2_ fluxes, perhaps caused by the heatwave in the late summer; hence, no quantitative assessment of the impacts of COVID-19 on ffCO_2_ can be made using the atmospheric CO_2_ data.

## DISCUSSION

We have used APO, a derived tracer that can separate natural and anthropogenic contributions to regional atmospheric CO_2_ variations, to quantify the reduction in ffCO_2_ associated with the COVID-19 lockdowns in the United Kingdom during 2020–2021. Detection of COVID-19 signals is only possible owing to the high frequency of the APO measurements (which produce continuous, hourly data) and when combined with a machine learning algorithm that accounts for the influence of atmospheric transport variability on the observed ffCO_2_. It should be noted that although the machine learning algorithm allows the comparison of ffCO_2_ in the atmosphere between different years, it does not account for how atmospheric mixing translates CO_2_ fluxes into atmospheric mole fractions. We also acknowledge that training a statistical model for predictive purposes using trending data is likely to be inappropriate unless the trend is first removed. For our study, while there has been a decreasing trend in U.K. ffCO_2_ emissions since 2010, this trend is not visible in the APO-based ffCO_2_ at WAO (as shown in Fig. 2), due to the dominating influence of atmospheric transport variability on the ffCO_2_ signal compared to a relatively smaller decreasing trend; thus, in our case, there is no requirement to account for any ffCO_2_ emissions trend before training.

It might be feasible to replicate our machine learning–based analysis using a discrete, low-frequency ffCO_2_ tracer, but only with much larger uncertainty, since the random forest algorithm relies on having a considerable number of high-frequency (e.g., hourly) ffCO_2_ values. In this case, the timely detection of COVID-19 signals at WAO is only made possible by the availability of a continuous ffCO_2_ tracer combined with a method to remove the effects of atmospheric transport on the ffCO_2_ signal, such as the machine learning algorithm we have applied here.

Our COVID-19 signal, detected directly from atmospheric measurements as a mean decrease of 0.7 ppm in daily observed ffCO_2_ from March to December 2020, is in broad agreement with three U.K. bottom-up emissions estimates, based on indirect energy and activity data. We refer to atmospheric measurement-based top-down estimates as direct and bottom-up estimates as indirect, because what matters from a climate change perspective is the change in radiative forcing in the atmosphere, caused by changes in atmospheric greenhouse gas concentrations, which top-down methods are able to measure directly. Our APO-based analysis is able to resolve the two U.K. lockdown periods individually, which are separated by a period of recovery during the summer of 2020 when lockdown restrictions were eased. This APO analysis, using data from a single U.K. measurement station, indicates that a network of continuous APO measurement sites would have strong potential for providing top-down estimates of ffCO_2_ emissions at regional scales, which corroborates the results of a recent modeling analysis (*42*). Furthermore, since the WAO APO data are measured and calibrated in situ and in real time, APO data could be highly beneficial in providing timely top-down ffCO_2_ estimates in the future.

The APO network of stations is currently sparse with few measurement sites ideally situated to capture anthropogenic emissions signals. Thus, using APO as a tool for top-down ffCO_2_ emissions quantification efforts at scale will require investment in precise and accurate atmospheric O_2_ and CO_2_ measurements, which are technically challenging, and improved knowledge of R_APO_ from emissions inventories.

## MATERIALS AND METHODS

### Measurements and calculations of CO_2_, O_2_, APO, and ffCO_2_[APO] at the WAO, United Kingdom

WAO is situated on the north Norfolk coast (53°N) in a rural part of the United Kingdom. Atmospheric O_2_ is measured every two minutes with a Sable Systems International Inc. “Oxzilla II” electrochemical fuel cell analyzer, and CO_2_ is measured with a Siemens Corporation “Ultramat 6E” nondispersive infrared analyzer (*43*). O_2_ measurements are reported on the Scripps Institution of Oceanography, USA O_2_ scale (*44*) and CO_2_ measurements are reported on the World Meteorological Organization CO_2_ X2007 scale (*45*). Atmospheric O_2_ measurements are reported as δ(O_2_/N_2_) ratios in per meg units, rather than mole fractions, because O_2_ is not a trace gas, and its mole fraction is therefore affected by small changes in other gases, such as CO_2_ (*36*).

APO is calculated from measurements of atmospheric O_2_ and CO_2_ (*33*) according to

$$\text{APO}\approx \delta (O_{2}/N_{2})+1.1/0.2094\times (350-{\text{CO}}_{2})$$ (2)

where the value of 1.1 denotes the mean −O_2_:CO_2_ molar ratio of terrestrial biosphere-atmosphere exchange (*34*), 0.2094 is the standard mole fraction of O_2_ in dry air, and 350 is an arbitrary reference value for CO_2_ in parts per million, based on the CO_2_ mole fraction of cylinders that define zero on the Scripps Institution of Oceanography calibration scale. By summing O_2_ and CO_2_ observations in this way, APO becomes invariant to terrestrial biosphere exchange by definition. Variability in APO therefore reflects mostly ocean-atmosphere exchange (on seasonal and long-term time scales) and fossil fuel combustion (on short- and long-term time scales). Our calculation of APO is approximate because it does not account for the influences of CH_4_ and CO (*33*), which have a negligible effect on APO at WAO.

The stoichiometry of O_2_ and CO_2_ exchange between the atmosphere and terrestrial biosphere has been shown to be robust on a global scale. Atmospheric O_2_ and CO_2_ observations from a range of locations sampling well-mixed tropospheric air have consistently found −O_2_:CO_2_ ratios to be within 1.10 ± 0.05, with very little temporal or spatial variability observed. An extensive range of surveys have found that all major organic pools on land have −O_2_:CO_2_ values ranging from 1.0 to 1.2 (*46*), while the −O_2_:CO_2_ exchange ratio of unpolluted air at WAO has previously been shown to lie within 1.10 ± 0.05 (*47*).

We calculate the regional fossil fuel component of atmospheric CO_2_ mole fractions (ffCO_2_) in units of parts per million at WAO using hourly averaged APO data as shown in Eq. 1. APO_BL_, the hourly APO “baseline” values (i.e., values that are representative of the well-mixed troposphere of the wider region), was determined using a statistical baseline fitting method (*48*), because there is not presently a suitable up-wind station with APO data from which we can obtain a measured baseline; thus, we sometimes obtain negative ffCO_2_ values. *R*_APO_ is the hourly APO:CO_2_ combustion ratio for fossil fuel emissions, calculated by converting −O_2_:CO_2_ molar ratios for fossil fuel combustion (α_F_) from the “COFFEE” (CO_2_ release and oxygen uptake from fossil fuel emission estimate) database (*35*) into −APO:CO_2_ molar ratios according to

$$R_{\text{APO}}=\alpha_{F}–1.1$$ (3)

where 1.1 is the −O_2_:CO_2_ molar ratio of terrestrial biosphere-atmosphere exchange as mentioned above. For our COVID analysis, we use a mean *R*_APO_ value at WAO of 0.37, obtained from STILT (*17*), which is run using gridded α_F_ values from the COFFEE database.

APO has historically been used to attribute variations in atmospheric O_2_ due to oceanic processes [e.g., (*33*, *37*, *49*, *50*)]; however, we estimate the effects of oceanic influences on our APO ffCO_2_ estimates to be minimal, because oceanic influences on APO mostly occur on seasonal or longer time scales, with short-term variations in APO dominated by fossil fuel emissions, and because the influence of short-term ocean-related variability on O_2_ at WAO has been estimated to be only ~6% (*51*); thus, the majority of oceanic influences in APO are incorporated into APO_BL_ and are excluded from ffCO_2_[APO].

### Calculation of hourly ffCO_2_[APO] uncertainties

We include here information about how to account for the sources of hourly uncertainty associated with each term in Eq. 1 as follows. Uncertainty in the APO data is calculated from typical ±1σ SDs in the hourly CO_2_ and O_2_ measurements at WAO during stable atmospheric conditions with low natural variability. In this manner, we incorporate uncertainty in analyzer performance and routine calibrations, as well as any uncertainty introduced by the measurement system, in pumping outside air from the tower, drying it, and passing it to the O_2_ and CO_2_ analyzers. Our APO data uncertainty also incorporates an estimate of the uncertainty associated with the −O_2_:CO_2_ molar ratio of terrestrial biosphere-atmosphere exchange, for which we use ±0.05 (*34*). We obtain a typical hourly APO uncertainty of approximately ±2 per meg or ±0.4 ppm equivalent units. Using a mean short-term range of observed APO variability of 49 per meg, the relative APO measurement uncertainty is therefore ±4.1%. The APO range is used rather than the mean APO value because APO is a quantity defined relative to an arbitrary reference.

Determination of a “true” atmospheric baseline concentration is complicated and poorly defined for almost all atmospheric greenhouse gases and pollutants. Ideally, one would use other stations to directly measure an appropriate baseline from clean-air/up-wind sites; however, it is rare that atmospheric measurement networks are sufficiently dense or optimized to allow this method of baseline determination. Since there is currently no upwind O2 measurement site for WAO, we use “rfbaseline,” a statistical baseline estimation technique with a smoothing window of approximately 1 week, which is fitted to the WAO APO data themselves and which tracks values deemed to be unaffected by local influences (*48*). Uncertainty of the baseline is estimated by recalculating ffCO_2_[APO] with varying “stiffness” in the fitting routine, using span values of 0.03, 0.06, and 0.12, representing smoothing windows of approximately 1, 2, and 4 weeks, respectively. The choice of baseline stiffness mostly affects the magnitude of the ffCO_2_ obtained, not the variability, which is determined by variability in the APO data. We estimate an APO_BL_ uncertainty of ±2.81 per meg, or ±0.6 ppm equivalent units, calculated from the mean differences between the 1- and 4-week smoothed baselines compared to the 2-week smoothed baseline. As a relative uncertainty, we find APO_BL_ to be ±28%, based on a mean short-term range of APO_BL_ variability of 10 per meg.

Uncertainty in the gridded fossil fuel emission ratio estimates of *R*_APO_ are not provided in COFFEE; instead, we use the ±1σ SD (i.e., the variability) around the mean STILT-COFFEE *R*_APO_ value of 0.37 as a proxy for uncertainty in *R*_APO_ at WAO, giving an absolute value of ±0.11. The relative uncertainty in *R*_APO_ is therefore ±31% (±1σ divided by the mean, multiplied by 100). The uncertainties of both α_F_ and the O_2_:CO_2_ molar ratio of terrestrial biosphere-atmosphere exchange are included in our total *R*_APO_ uncertainty as per Eq. 3.

Converting the absolute uncertainty of each term in Eq. 1 into relative uncertainties allows the total hourly ffCO_2_[APO] uncertainty to be calculated using the rules of error propagation. We estimate a mean hourly ffCO_2_[APO] uncertainty of ±42%, which is dominated by our estimate of uncertainty in *R*_APO_, followed by our estimate of uncertainty in APO_BL_. At WAO, during the period 2010–2021, the mean ffCO_2_[APO] detected is 3.4 ± 1.4 ppm. We reiterate that our estimate of *R*_APO_ uncertainty, while being the largest term, is only a proxy for the actual uncertainty owing to a lack of available information and that future work on using APO to quantify ffCO_2_ should focus on refining *R*_APO_ values and their uncertainties. Uncertainty in APO_BL_ can likely be reduced with denser networks of APO data, which may allow APO_BL_ to be calculated from other sites, instead of having to rely on statistical fitting methods that likely have a higher uncertainty.

### Machine learning analysis and COVID-19 signal detection

To account for the influence of atmospheric transport on our ffCO_2_[APO] dataset, we use the “rmweather” R package (version 0.1.51) (*52*, *53*), which has previously been used with air pollution datasets (*39*, *53*, *54*). The rmweather package uses random forest, an ensemble decision tree machine learning method (*55*) that splits observations using a binary algorithm into two homologous groups, known as branches, repeating the process until the “tree” is fully grown [“node purity” is achieved (*54*)]. Decision trees are prone to overfitting (*56*), but random forest mitigates this by growing many individual decision trees from a training set in a process called bagging (bootstrap aggregation), which creates a forest of decorrelated trees, since each has been grown on different subsets of the training set.

Using rmweather, we train a random forest model of 300 trees at WAO for the period 2010–2019 using 10 independent variables: hourly meteorological observations (wind speed, wind direction, air temperature, relative humidity, and atmospheric pressure), which are measured in situ at WAO; temporal factors (day of the year, day of the week, and hour of the day); hourly atmospheric radon-222 activity, a tracer for atmospheric mixing that has been measured at WAO since April 2018 using an Australian Nuclear Science and Technology Organisation monitor (*57*); and hourly 24-hour-long HYSPLIT (https://ready.noaa.gov/HYSPLIT.php) model (*58*) backward-run trajectories, clustered into eight groups (see fig. S5) using *k*-means clustering and the openair package in R (*59*) (https://davidcarslaw.github.io/openair/). For the dependent variable, we use hourly ffCO_2_[APO], calculated using Eq. 1. Meteorological data are cross-checked against an independent (but colocated) dataset operated by the U.K. Met Office as a quality control measure. The training set consists of 80% of the data, with 20% reserved for testing.

Performance of the trained model was assessed for bias and goodness of fit, as shown in Fig. 4. We use the model, trained on the 2010–2019 data to predict the counterfactual ffCO_2_ that would have been observed at WAO during the time period 1 February 2020 to 31 January 2021, if the COVID-19 pandemic had not occurred. This counterfactual prediction is then compared to the observed ffCO_2_[APO] values over the same time period to estimate the impact of COVID-19 lockdown measures on ffCO_2_ at WAO. We calculate the relative percent change in emissions, *E*, by taking the ratio of the cumulative ffCO_2_[APO] and cumulative ffCO_2_ counterfactual signals shown in Fig. 3B according to

$$E=(1-\frac{{\text{ffCO}}_{2}{\left[\text{APO}\right]}_{\text{cumulative}}}{{\text{ffCO}}_{2}{\left[\text{counterfactual}\right]}_{\text{cumulative}}})\times 100\%$$ (4)

As mentioned in the “Analysis of uncertainties” section (see Results), the uncertainty range we report on the COVID-19 ffCO_2_ relative emissions reductions shown in Fig. 3B is based solely on the uncertainty associated with the machine learning algorithm (calculated from the difference in model performance between training and predictive results), since the APO-based uncertainties cancel.

### Comparison with bottom-up inventory estimates

We compare our COVID-19 signal detected from ffCO_2_[APO] data to three bottom-up inventory estimates for the United Kingdom, which quantify COVID lockdown emissions reductions by comparing 2020 emissions to those from 2019. These are the following: inland energy consumption statistics from the U.K. BEIS, which we convert to CO_2_ emissions estimates in units of megatons using coal, gas, and oil conversion factors and by tuning to annual emissions from previous years; an updated version (March 2021) of the UEA estimate (*3*), based on a combination of energy, activity, and policy data; and an estimate from Carbon Monitor, based on fuel consumption and activity data (*4*). Only the Carbon Monitor emissions have daily resolution; the UEA and U.K. BEIS estimates are monthly. We exclude the 29 February from all estimates (bottom-up and top-down) to enable a comparison of leap years to nonleap years. At the time of publication, only the UEA estimate fully includes emissions from IAS.

## Supplementary Materials

### This PDF file includes:

Appendix SA. Detailed derivation of ffCO_2_[APO] Figs. S1 to S7
