# Supplementary material for: Novel quantification of regional fossil fuel CO2 reductions during COVID-19 lockdowns using atmospheric oxygen measurements
Source: Sci Adv. 2022 Apr 22;8(16):eabl9250. doi: 10.1126/sciadv.abl9250 (PMC9032948; doi:10.1126/sciadv.abl9250)
Supplement: Supplementary file 1 — Appendix SA. Detailed derivation of ffCO2[APO] Figs. S1 to S7 [file sciadv.abl9250_sm.pdf]

Science Advances
AAAS

Supplementary Materials for

Novel quantification of regional fossil fuel CO_2 reductions during COVID-19 lockdowns using atmospheric oxygen measurements

Penelope A. Pickers*, Andrew C. Manning, Corinne Le Quéré, Grant L. Forster, Ingrid T. Luijkx, Christoph Gerbig, Leigh S. Fleming, William T. Sturges

*Corresponding author. Email: p.pickers@uea.ac.uk

Published 22 April 2022, *Sci. Adv.* **8**, eabl9250 (2022)
DOI: 10.1126/sciadv.abl9250

This PDF file includes:

Appendix SA. Detailed derivation of $ffCO_2$[APO]
Figs. S1 to S7

Appendix SA. Detailed derivation of $ffCO_2$[APO].

$\Delta CO_2 = F - O - L$

where ΔCO_2 is the change in atmospheric CO_2, F is CO_2 from fossil fuel emissions (a net source of CO_2 to the atmosphere), O is the net CO_2 exchange between the ocean and the atmosphere (a sink), and L is the CO_2 exchange between the land biosphere and the atmosphere (also a sink; includes fluxes from land-use change).

$\Delta O_2 = \alpha_F*F - \alpha_L*L$

where ΔO_2 is the change in atmospheric O_2, F and L are CO_2 fluxes as defined above, α_F is the mean oxidative ratio ($-O_2:CO_2$) of global fossil fuel emissions, and α_L is the mean oxidative ratio of global terrestrial biosphere-atmosphere exchange ($-O_2:CO_2$).

Considering the CO_2 and O_2 mass balance over a regional domain:

$CO_2 = H(F) - H(L) - H(O) + CO_{2\text{-}BL}$

where H is a transport operator and $CO_{2\text{-}BL}$ is the baseline CO_2 concentration

$O_2 = \alpha_F*H(F) + \alpha_L*H(L) + O_{2\text{-}BL}$

where $O_{2\text{-}BL}$ is the baseline O_2 concentration. It is assumed there is no regional net ocean flux for O_2 because CO_2 and O_2 ocean-atmosphere exchange is decoupled, owing to the fact that CO_2 dissociates in seawater but O_2 does not.

As defined in Equation 1:

$APO = O_2 + \alpha_L*CO_2$

Therefore,

$APO = (\alpha_F+\alpha_L)*H(F) - \alpha_L*H(O) + O_{2\text{-}BL} + \alpha_L*CO_{2\text{-}BL}$

For APO_{BL} (i.e. the signal coming from the ocean onto the land) F = 0 so:

$APO_{BL} = -\alpha_L*H(O) + O_{2\text{-}BL} + \alpha_L*CO_{2\text{-}BL}$

Thus,

$ffCO_2[APO] = (APO - APO_{BL})/(R_{APO}) = (APO - APO_{BL})/(\alpha_F+\alpha_L) = H(F)$

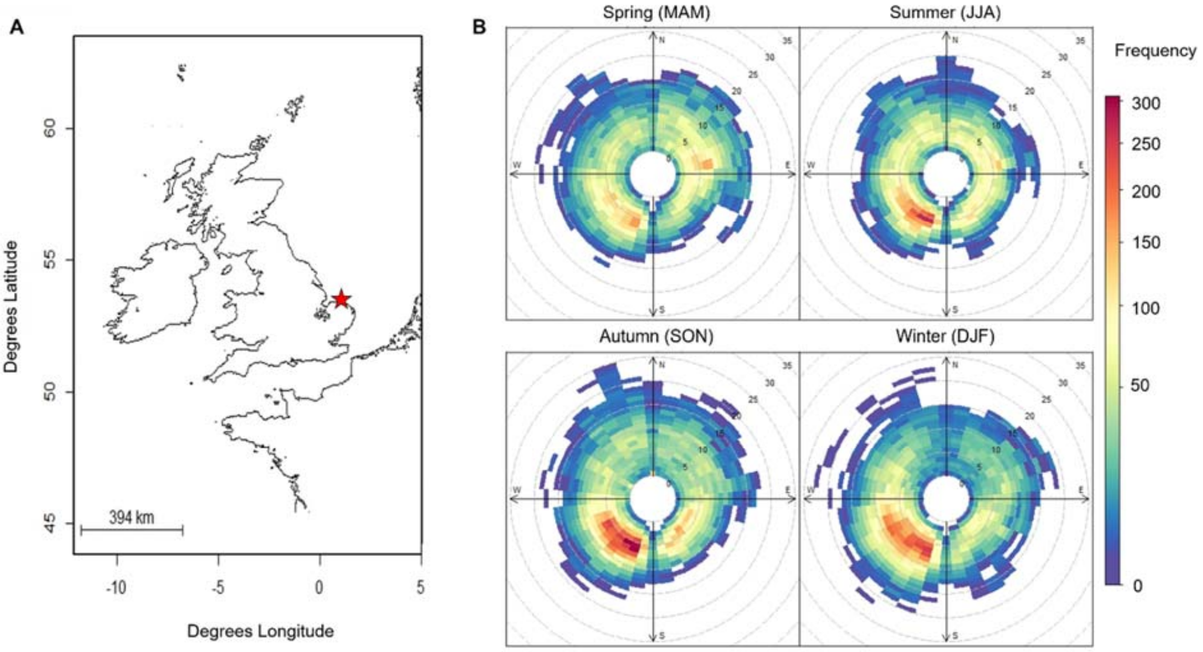

Figure S1. Supplementary plots of WAO. (A) Map of the UK, showing the location of Weybourne Atmospheric Observatory on the north Norfolk Coast (red star). (B) Polar frequency plot showing wind speed (in m s^{-1}) and wind direction for May 2010 – Jan 2021, split by season. South-westerly winds dominate during all seasons, except in spring when there is a greater contribution from the north-east sector.

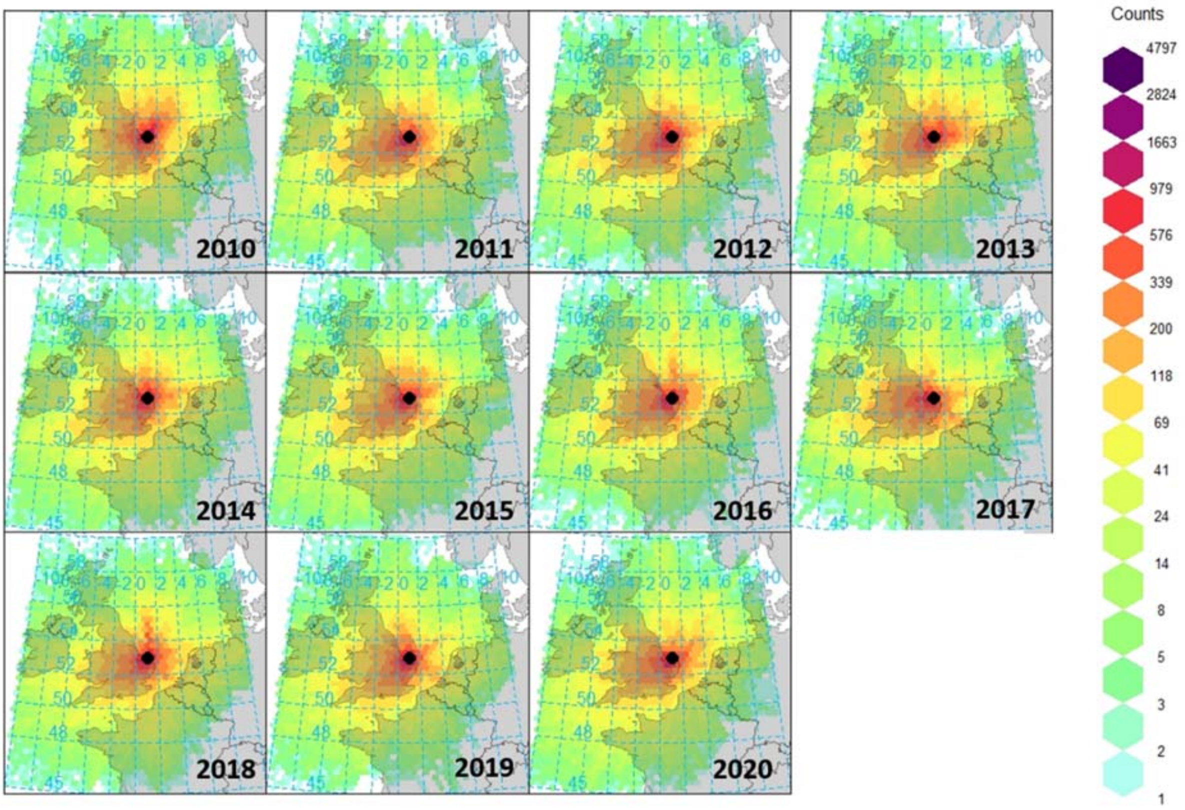

Figure S2. WAO footprint for each year 2010-2020, from 3-hourly HYSPLIT trajectories, binned for each map grid box. The WAO annual footprint exhibits interannual variability, which affects the concentrations of atmospheric species measured at the site.

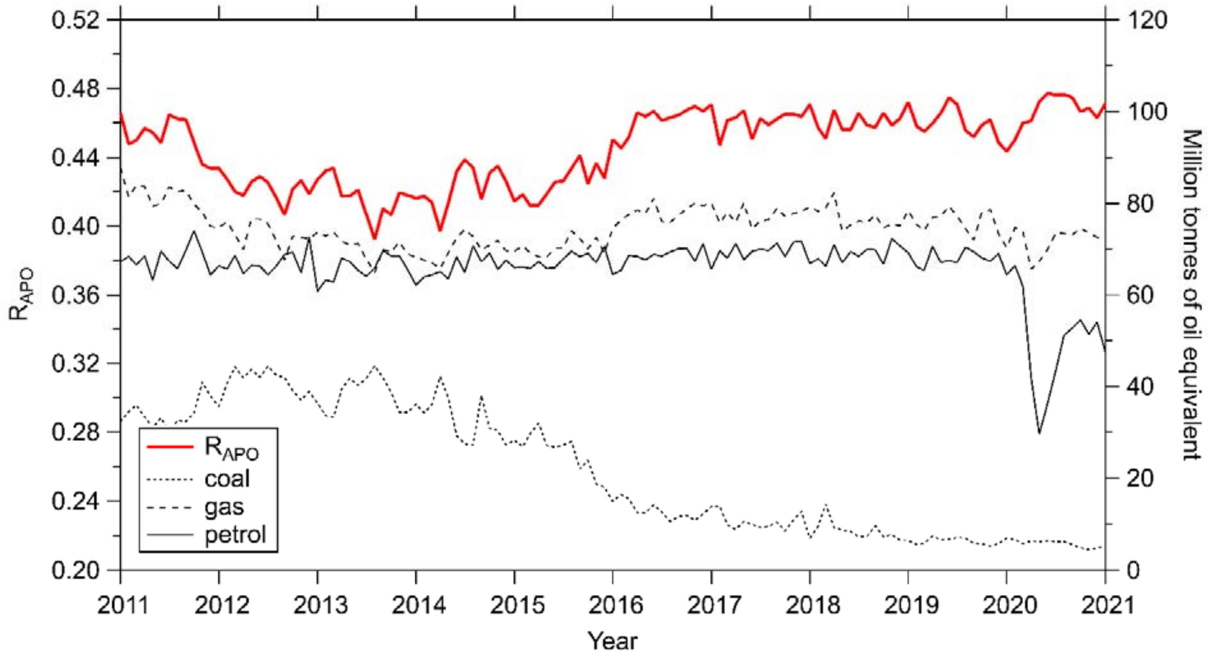

Figure S3. R_{APO} values calculated from the UK BEIS energy trends data for the period January 2011 to January 2021 inclusive. R_{APO} is shown by the red line (left axis) with coal, gas and petrol shown by the black lines (right axis). R_{APO} increases by 0.01 after March 2020, during the COVID-19 lockdown periods. The earlier change in R_{APO} during 2012-2016 is caused by a reduction in coal usage, which is later compensated by an increase in gas usage. The reason for the difference between the mean R_{APO} value determined from the BEIS data compared to the value we obtained from STILT (BEIS is about 0.08 higher) is not currently known.

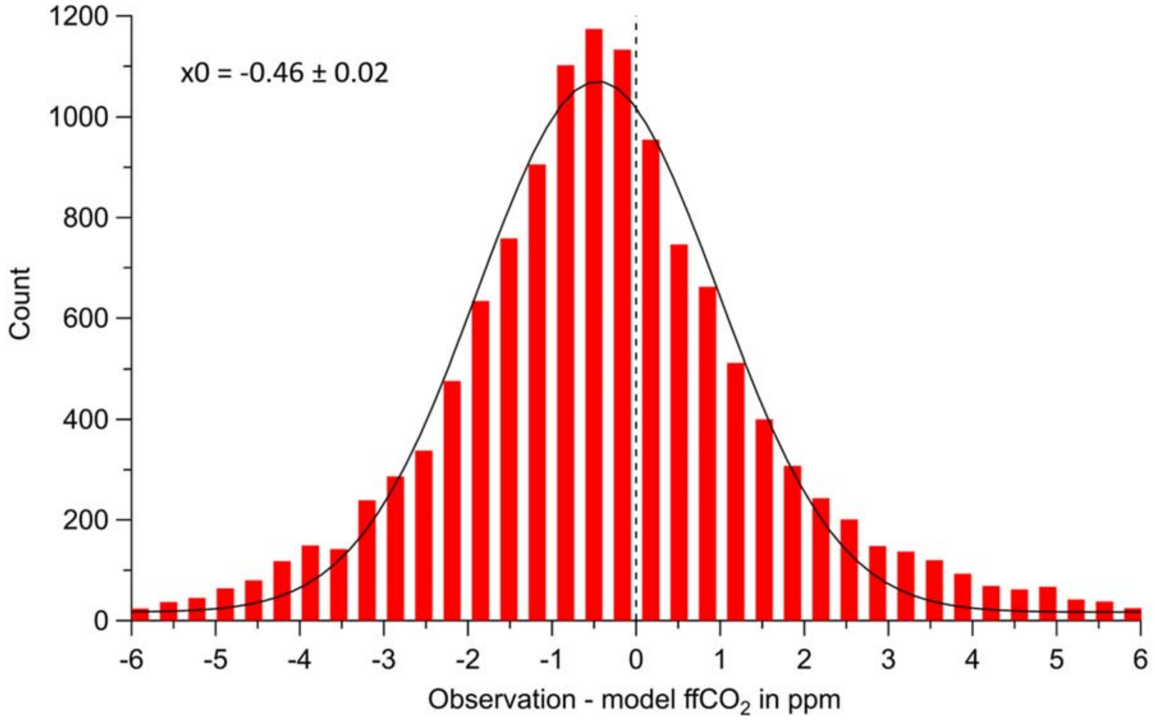

Figure S4. Histogram of the observation – model differences shown in Figure 4. The plot shows data from the model test set only, which are withheld from model training. The black solid line is a gaussian fit of the binned differences. Only differences in the range -6 to 6 ppm are shown for clarity (the counts for larger differences are all very low). x0 denotes the x-axis position of the peak of the gaussian fit ± 1σ standard deviation. The value differs from that of Figure 4A in the main text, in part because of the binning process, and in part because the observation-model differences have a slightly non-normal distribution.

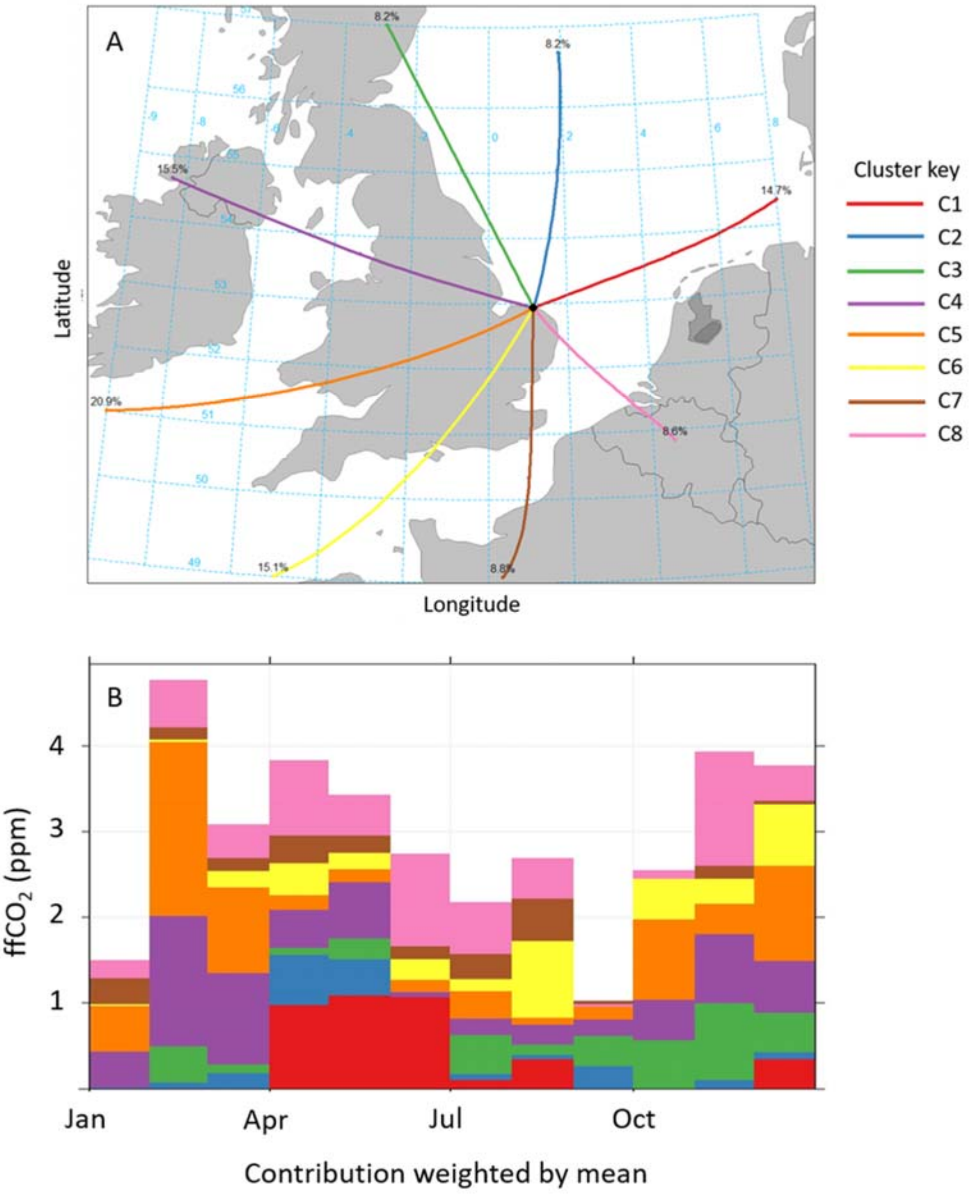

Figure S5. Air mass backwards trajectory analysis for WAO. (A) Clustered air mass backwards trajectories at WAO for the years 2010-2020. These clusters were used as one of the ten independent variables in the machine learning analysis (see Methods). (B) Time proportion plot for the year 2020, showing the contribution of each cluster per month. UK-originating air masses dominate the trajectories of WAO during most months, with some significant contributions from the North Sea (especially during the late spring/early summer) and the European continent.

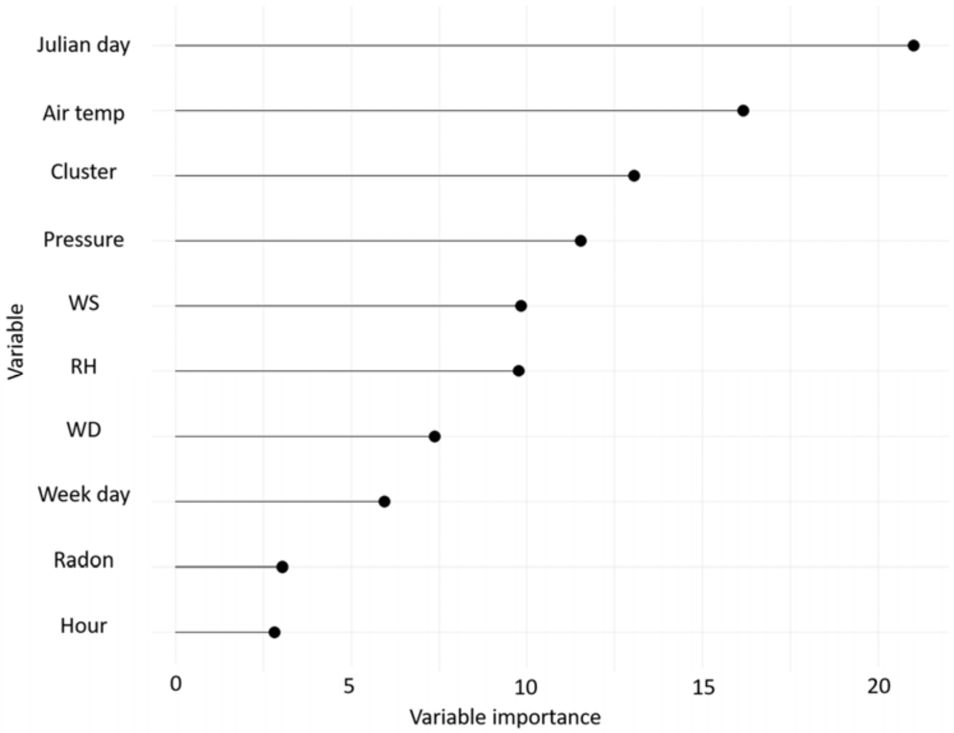

Figure S6. Variable importance plot of the 10 independent variables of the trained Random Forest model. The variable importance, shown on the x axis, is defined as "the permutation importance differences of the predicted errors" and is unitless (*39*). Variable importance provides a measure of the strength of influence of a variable on prediction and is ranked for the 2010-2019 period. Thus, Radon-222, for which the data only exist at WAO since April 2018, ranks low in this plot but is ranked much higher when the model is trained using only 2018-2019 data.

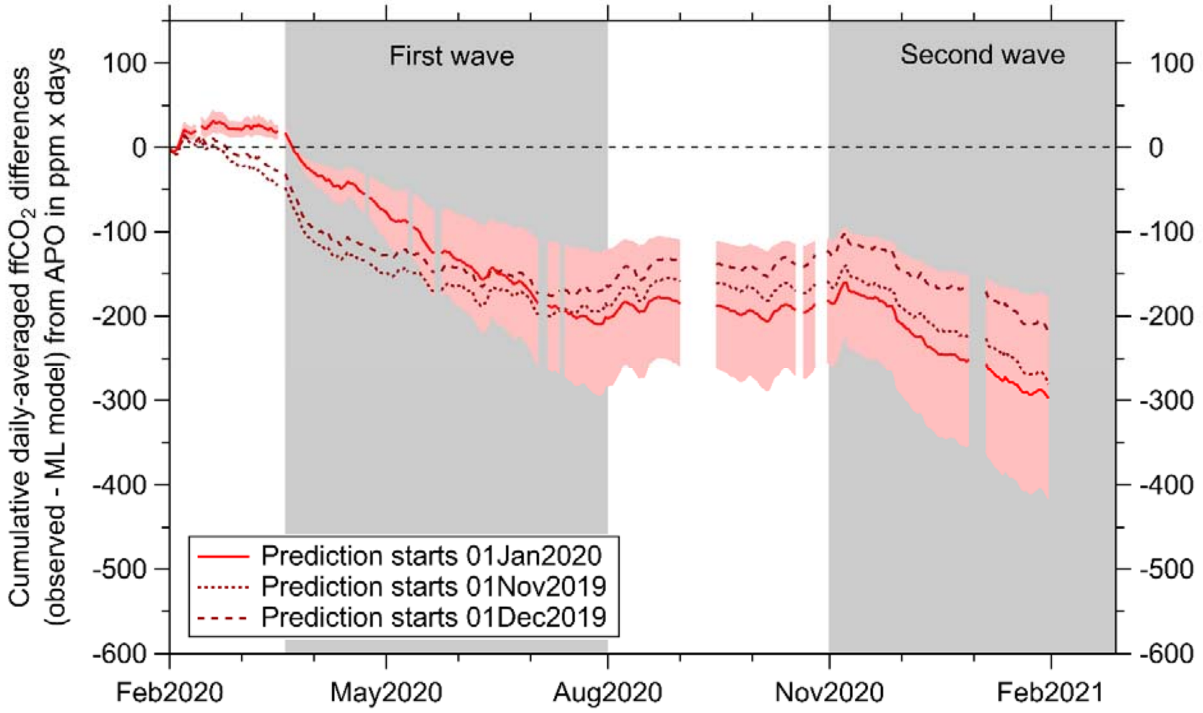

Figure S7. Sensitivity of the Random Forest prediction to different start dates: 01Jan2020 (solid red line and shaded uncertainty, which are the same as shown in Figure 3B in the main text); 01Nov2019 (dotted burgundy line); and 01Dec2019 (dashed burgundy line). The cumulative ffCO_2 differences of all three predictions are relative to 01Feb2020. Except for during the first few months, the differences in the results between the three predictions are within the uncertainties.
